# Primary closure versus secondary healing of pin sites after the removal of provisional external fixator: a systematic review and meta-analysis

**DOI:** 10.1186/s12891-025-09288-2

**Published:** 2025-11-24

**Authors:** Hamed Tayyebi, Michael McKee, Niloofar Dehghan

**Affiliations:** 1 University of Arizona College of Medicine Phoenix – Phoenix, Arizona, USA; 2 The CORE Institute, Phoenix, AZ USA

**Keywords:** External fixation, Pin entry site, Infection, Primary closure, Secondary healing

## Abstract

**Background:**

External fixators are commonly used for managing complex fractures, but they are associated with the risk of pin site infections. Pin site management following external fixator removal can be performed using either primary closure or secondary healing. The relative efficacy and safety of these approaches, particularly in terms of infection rates, remain uncertain.

**Objective:**

This systematic review and meta-analysis aimed to evaluate the infection rates of pin entry sites following the removal of the temporary external fixator, comparing primary closure and secondary healing methods.

**Methods:**

We conducted a systematic review and meta-analysis of studies comparing infection rates between primary closure and secondary healing after external fixator removal. Eligible studies included randomized controlled trials (RCTs), cohort studies, and other observational studies. Data were extracted on patient demographics, infection rates, and risk factors. The primary outcome was the rate of pin site infection, and data were synthesized using Odds Ratios (OR) with 95% confidence intervals (CI).

**Results:**

A total of three studies (one RCT and two cohort studies) were included in the analysis, with 738 patients. Pooled analysis showed significantly lower infection rates in primary closure compared to secondary healing (OR: 0.375, 95% CI: 0.195–0.724; I² = 0.0%, *P* = 0.003). However, there was considerable heterogeneity among the studies, which may have contributed to the inconsistent results. Factors such as duration of external fixation, anatomical site of fixation (upper vs. lower extremity), and comorbidities may have influenced the infection rates.

**Conclusion:**

This meta-analysis demonstrated that primary closure of pin sites was associated with a lower risk of infection compared with secondary healing, based on studies with a maximum external fixation duration of 21 days. However, future well-designed, homogeneous research is needed to better understand the impact of these two wound management approaches on pin site infections.

## Introduction

External fixators are orthopedic devices widely used for the provisional management of complex open fractures [1]. Despite their benefits in providing stable fixation, the use of external fixators is associated with potential complications, including pin entry site infection [1].

After the removal of an external fixator, pin site wounds can be managed using one of two approaches: primary closure or secondary healing [2–4]. Primary closure involves suturing the pin site immediately to accelerate wound healing. However, this method has raised concerns about the risk of trapping contaminants within the closed environment, which may lead to an increased risk of infection. In contrast, secondary healing allows the pin sites to close naturally without surgical intervention, relying on the body’s intrinsic healing mechanisms. Although this method generally takes longer, it is thought to minimize infection risks associated with closure [2–4]. Consequently, most orthopedic surgeons opt for secondary healing, leaving the pin sites open to reduce infection risk, albeit at the expense of greater patient discomfort [5]. This preference appears to be rooted in longstanding surgical dogma rather than robust evidence, underscoring the need for high-quality research to determine whether this practice should be reconsidered.

Some recent studies have compared infection rates between primary closure and secondary healing of pin sites following the removal of the temporary external fixator [6–8]. Despite these efforts, the choice between the two strategies remains a subject of debate, with no clear consensus established. This highlights the need for high-quality evidence synthesis to clarify the outcomes of primary closure versus secondary healing and support informed clinical decision-making. This systematic review and meta-analysis aim to evaluate the safety of these approaches, with a particular focus on infection rates [9].

## Materials and methods

This systematic review and meta-analysis were conducted in accordance with the Preferred Reporting Items for Systematic Reviews and Meta-Analyses (PRISMA) statement [10]. Eligible studies assessing the impact of primary closure and/or secondary healing on infection rates at pin entry sites following external fixator removal were included in this analysis.

### Eligibility criteria

Studies of any design, including randomized clinical trials, case-control studies, cohort studies, and observational studies, were considered for inclusion. The eligibility criteria were as follows: Human studies, studies reporting infection rates at pin entry sites following the removal of temporary external fixators, studies providing separate infection rates for primary healing and/or secondary closure, and studies written in English. Studies that did not report the raw number of infections (e.g., those using alternative measures like odds ratios) were deemed ineligible, as their data could not be analyzed using the meta-analysis software. In cases where data were unclear or incompletely reported, we attempted to contact the corresponding authors of the included studies via email. No additional data were obtained despite these efforts.

### Information sources and search strategy

This systematic review followed PRISMA guidelines. A comprehensive literature search was conducted across the following electronic databases: PubMed, Scopus, Web of Science, EMBASE, and the Cochrane Library, from inception to January 14, 2025, without any restrictions on publication date or language. The search strategy combined both free-text terms and controlled vocabulary (e.g., MeSH and Emtree terms) where applicable. The main search terms included: “external fixator”, “pin entry site”, “pin insertion site”, “pin tract”, “infection”, “primary closure”, and “secondary healing”, using appropriate Boolean operators (AND/OR).

In addition, a manual search of relevant conference proceedings was performed, including the Orthopaedic Trauma Association (OTA) website and conference abstracts to ensure inclusion of the most recent and unpublished studies. The reference lists of all included articles and previous systematic reviews were also manually screened to identify any additional relevant studies.

Two independent reviewers (HT and ND) conducted the database and manual searches. Any discrepancies were resolved through consensus.

Although a formal review protocol was not registered prospectively on PROSPERO, the review methodology and search strategy were clearly defined and followed rigorously throughout the study process.

### Data extraction

Two reviewers (HT and ND) independently extracted data from the eligible studies, including study characteristics (first author’s name and year of publication), patient demographics (age, gender, and follow-up duration), comorbidities (such as diabetes), details of external fixator use (location on upper or lower limb and duration of implementation), the number of patients whose pin entry sites were managed with primary healing or secondary closure, and the infection rates reported for each group.

### Risk of bias assessment

The New Castle-Ottawa Quality Assessment Scale (NOS) was used to evaluate the risk of bias in cohort studies [11], while the Revised Cochrane Risk-of-Bias Tool for Randomized Trials (RoB 2) was applied to assess bias in randomized controlled trials [12]. The NOS evaluates the risk of bias across three main domains: selection of study groups, comparability of groups, and exposure or outcome assessment. It assigns a score ranging from 0 to 9, with higher scores indicating a lower risk of bias. The RoB 2 evaluates five key domains in randomized trials: randomization process, deviations from intended interventions, missing outcome data, measurement of outcomes, and selection of reported results. Each domain is rated as low, high, or unclear risk, and the overall risk of bias is determined based on these individual ratings. Two authors (HT and ND) independently assessed the quality of the studies, and any disagreements were resolved through a consensus discussion.

### Primary outcome

The primary outcome of interest was the rate of pin-entry site infection, managed through either primary healing or secondary closure, following the removal of external fixators. Pin-site infections were defined as clinical signs of infection, including local redness, swelling, and/or purulent discharge requiring surgical intervention or antibiotics, either orally or intravenously.

### Data synthesis and statistical analysis

Data synthesis and statistical analysis were done using the completely open-source, cross-platform software for advanced meta-analysis Open Meta[Analyst], Centre for Evidence-Based Medicine at Brown University [13]. Odds Ratios (OR) with 95% confidence intervals (CI) were calculated for the infection rate based on the pin-entry site management approaches, and the Forest plot was depicted accordingly. *P* < 0.5 was considered statistically significant.

## Results

### Study selection

**Fig. 1:**
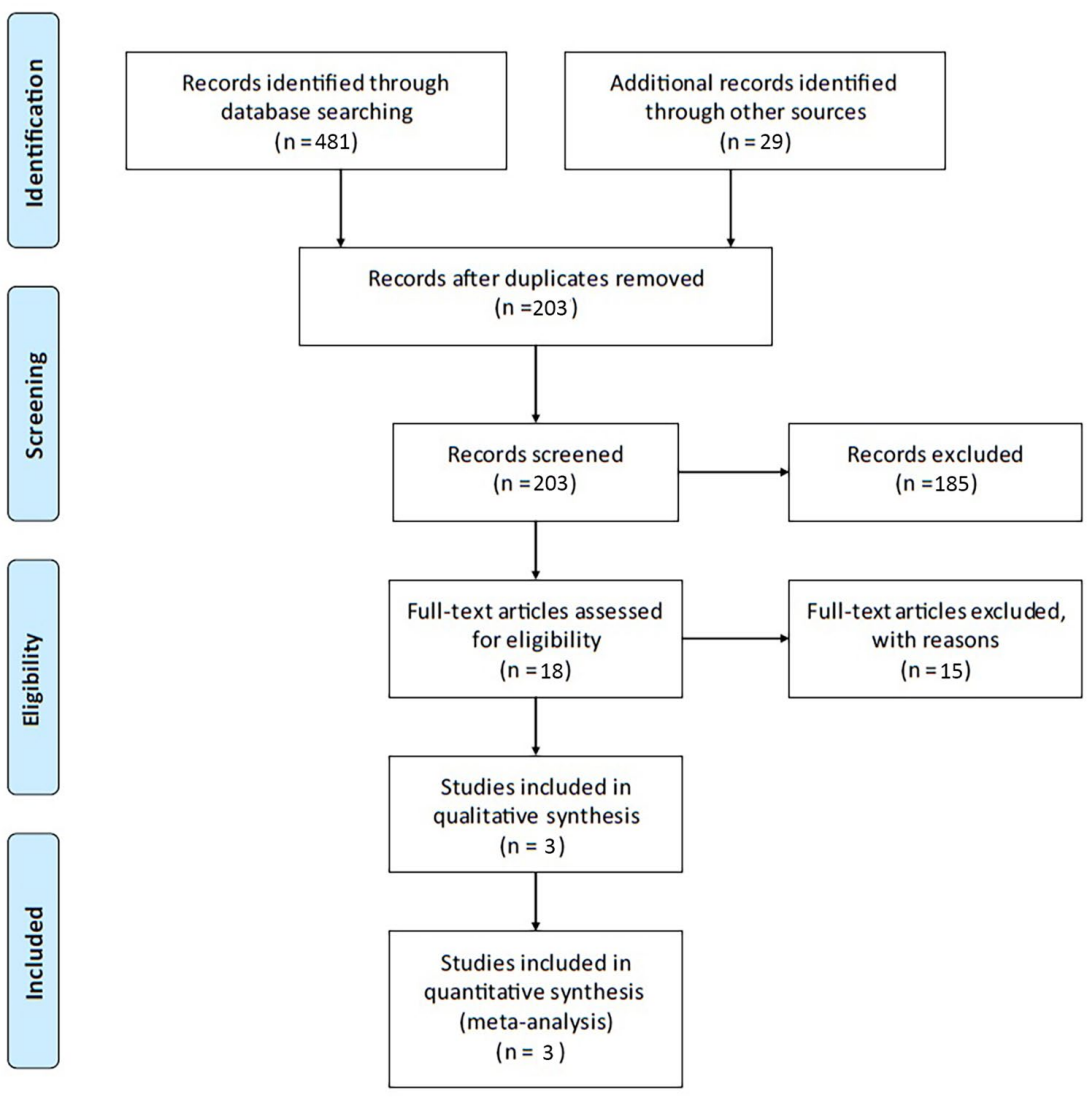
Flow diagram of the study selection

The database search identified 510 articles. After removing duplicates (*n* = 307), 203 unique manuscripts remained for title and abstract screening. This process resulted in the exclusion of 185 articles, leaving 18 manuscripts for full-text review. Following a detailed review of the full texts, 15 additional manuscripts were excluded for not reporting data separately for the two approaches, leaving only three manuscripts eligible for inclusion in the final analysis (Fig. 1). These comprised one randomized controlled trial and two cohort studies (Table 1). The cohort studies were performed in the United States [7, 8], while the RCT was done in Switzerland [6].

**Table 1:** Characteristic features of the eligible studies

Study	Year of publication	Study design	Number of patients	Male	Mean Age (year)	Diabetes	Primary/Secondary closure	Upper/lower extremity	Mean Fixation duration	Infection
Tillmann et al.	2023	RCT	70	44%	55	4%	123/118 pins	83/158	6 days	0
Brodell et al.	2024	Retrospective cohort	256	75%	49	12%	143/113 patients	0/256	12.2 days	Primary:0.5% Secondary: 1.5%
Sakar et al.	2024	Retrospective cohort	412	57%	53	18%	254/158 patients	22/390	21 days	Primary: 5.1% Secondary: 12.7%

Overall, 738 patients from three studies were included in the analysis. In the RCT by Tillmann et al. [6], all investigations were conducted within a single patient group, with only the management of pin sites being randomized. Specifically, the pin sites were managed alternately as either primary closure or secondary closure. The male sex ranged from 44 to 75% in the studies. The mean age of the patients ranged from 49 to 55 years. The rate of diabetic patients ranged from 4 to 18%. Brodell et al. [7] only included the lower extremities, while the other two studies [6, 8] included both the lower and upper extremities in the investigation.

The external fixator was used for damage control only, not for definitive treatment. The duration of external fixator implementation ranged from 6 to 21 days. Self-drilling or self-taping Schanz screws were used for all patients.

In the study by Tillmann et al. [6]., no pin site infection was recorded. In the study by Brodell et al. [7], pin site infection was recorded in 2 (0.5%) patients managed with primary closure and 5 (1.5%) patients managed with secondary healing. In the study by Sekar et al. [8], pin site infection was recorded in 13 (5.1%) patients who were managed with primary closure versus 20 (12.7%) patients who were managed with secondary closure.

### Pooled analysis of the infection rate

**Fig. 2:**
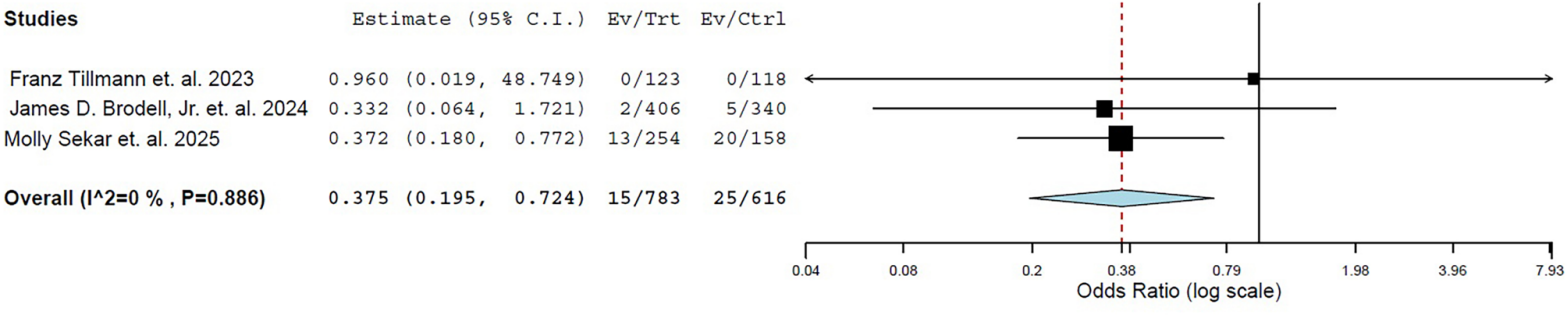
Forest plot comparing the rate of pin site infection between the primary closure and secondary healing

A pooled analysis showed a significantly lower rate of infection in pin entry sites managed with primary closure compared to those managed with secondary healing (OR: 0.375, 95% CI: 0.195–0.724; I^2^ = 0.0%, *P* = 0.003). The Forest plot of this comparison is demonstrated in Fig. 2.

### Risk of bias assessment

The RCT by Tillmann et al. [6] was assessed as having a low risk of bias based on the RoB 2 tool. The two cohort studies [6, 8] received an overall risk of bias score of 7 out of 9 on the Newcastle-Ottawa Scale (NOS), indicating a minimal risk of bias. Detailed results of the risk of bias assessment are presented in Tables 2 and 3.

**Table 2:** Risk of bias assessment using revised Cochrane risk-of-bias tool for randomized trials

Study	Risk of bias arising from randomization process	Risk of bias due to deviation from the intended intervention	Missing outcome data	Risk of bias in measurement of the outcome	Risk of bias in selection of the reported result	Overall risk of bias
Franz Tillmann et al. 2023 [6]	*	*	*	*	*	LOW

**Table 3:** Risk of bias assessment using new Castel-Ottawa outcome quality assessment scale for cohort studies

Study	Selection	Compatibility	Outcome	Overall rating
James D. Brodell, Jr. et al.	***	**	**	7 of 9
Molly Sekar. et al.	***	**	**	7 or 9

## Discussion

In this systematic review and meta-analysis, we examined the impact of primary healing versus secondary closure on the infection rate at pin entry sites following external fixator removal. The analysis included three studies: one RCT and two cohort studies. The pooled data analysis revealed a significantly lower rate of infection in pin entry sites managed with primary closure compared to those managed with secondary healing.

The efficacy and safety of primary closure versus secondary healing have been a longstanding topic of debate, with numerous studies exploring and discussing this issue in different conditions [14–19]. However, their application, specifically following the removal of an external fixator, has only recently come under investigation. The RCT by Tillmann et al. demonstrated significantly faster wound healing with primary closure (median of 2 versus 6 weeks), while other outcomes, such as infection rate, pain, and patient satisfaction, showed no significant differences [6]. In the retrospective study by Brodell et al., secondary healing was identified as a risk factor for surgical site infections, with patients managed by this approach having a notably higher risk, although non-significantly [7]. The retrospective study by Sekar et al. reported a significantly higher infection rate in patients managed with secondary healing, contrasting with the traditional belief that primary closure is associated with a higher risk of pin site infection [8]. Similarly, the present meta-analysis showed that primary closure of the pin entry site could reduce the risk of pin tract infection.

The inconsistent results of earlier studies could be attributed to significant heterogeneity among the studies. Several factors have been reported to affect the risk of pin site infection after the removal of external fixator, including type and grade of injury [20], male gender [21], smoking [21], diabetes [21], and other comorbidities [22]. Increased duration of pin fixation has also been associated with a higher rate of pin site infection [23]. The inconsistent results across studies may be explained by differences in risk factors for pin site infection. In the RCT by Tillmann et al. [6], both wound healing approaches were evaluated within the same patients, eliminating the influence of patient-related factors. However, the duration of external fixation in this study was significantly shorter compared to the two cohort studies (6 days versus 12.2 and 21 days), which may have influenced the outcomes. Additionally, the proportion of male patients and those with diabetes differed substantially between the study groups. Moreover, smoking history, a known risk factor for infection, was not reported in the cohort studies. The distribution of lower and upper extremity injuries also contributed to the heterogeneity. While Brodell et al. included only lower extremity injuries, the other two studies incorporated a mix of upper and lower extremity injuries. The type of pins and the technique used for pin insertion can also influence the rate of infection and should be given more consideration in future studies [24]. Earlier studies have indicated that excessive heat generation from high-speed drilling, insufficient cooling, or multiple insertion attempts may predispose the pin tract to bacterial colonization and infection [25, 26]. To minimize the infection risk, careful pre-drilling with a sharp bit at controlled speeds, continuous saline irrigation to reduce thermal damage, and secure bicortical placement of the pin without undue loosening or instability is recommended [25, 26].

This meta-analysis had several limitations. The primary limitations were the small number of available studies and the considerable heterogeneity between them. Although the statistical measure of heterogeneity (I²) in our meta-analysis was 0%, indicating no significant heterogeneity in the pooled infection rates, we observed substantial clinical and methodological heterogeneity among the included studies. This included variations in patient comorbidities such as diabetes, duration of external fixation, anatomical site of fixation (upper vs. lower extremity), and differences in outcome reporting (per pin vs. per patient). These factors, while not reflected in statistical heterogeneity, could still influence outcomes and limit the comparability across studies. Therefore, our reference to ‘significant heterogeneity’ was intended to reflect these real-world clinical differences rather than statistical inconsistency alone.

While interpreting the results of this meta-analysis, it is important to consider differences in how infection rates were reported across studies. Study by Tillmann et al. reported pin site infections based on the total number of pins, the other study by Brodell et al. reported pin site infections based on the total number of pins and number of the patients. However, the study by Sekar et al. reported infection rates per patient and did not provide data on the total number of pins used. This inconsistency in outcome reporting limited our ability to perform a uniform per-pin analysis across all studies. Therefore, further well-designed, homogeneous studies are needed to more precisely determine whether primary closure or secondary healing is associated with a higher risk of pin site infection.

## Conclusion

This meta-analysis demonstrated that primary closure of pin sites was associated with a lower risk of infection compared with secondary healing, based on studies with a maximum external fixation duration of 21 days. While the statistical heterogeneity was low, clinical and methodological differences across studies remain important limitations. Future studies would benefit from more consistent reporting of key factors that influence infection risk, including the technique of pin insertion, the presence of factors affecting infection risk such as diabetes and smoking, anatomical location (upper vs. lower extremity), type of injury (open vs. closed), and the number of pins used. Standardization of these variables will help optimize homogeneity, facilitate stronger pooled analyses, and provide clearer guidance for clinical practice.

## Data Availability

All data generated or analysed during this study are included in this published article and its supplementary files.
